# Nonpneumatic Antishock Garment Combined with Bakri Balloon as a Nonoperative “Uterine Sandwich” for Temporization of Massive Postpartum Hemorrhage from Disseminated Intravascular Coagulation

**DOI:** 10.1155/2015/124157

**Published:** 2015-01-08

**Authors:** Andrea Jelks, Monica Berletti, Liliana Hamlett, Michele Hugin

**Affiliations:** Santa Clara Valley Medical Center, Department of Obstetrics and Gynecology, San Jose, CA 95128, USA

## Abstract

Disseminated intravascular coagulation (DIC) is an uncommon but potentially catastrophic complication of postpartum hemorrhage. We describe two cases of massive postpartum hemorrhage complicated by DIC that were successfully temporized with combined use of the Bakri balloon and nonpneumatic antishock garment (NASG) during massive transfusion. In the first case, a healthy, term gravida underwent emergent cesarean for fetal bradycardia during labor induction. 10 minutes after completion of surgery, brisk vaginal hemorrhage of nonclotting blood from fulminant DIC resulted in maternal shock. A Bakri balloon and NASG were placed during massive transfusion, resulting in rapid maternal stabilization. In the second case, a healthy, term gravida suffered an amniotic fluid embolism during labor requiring emergent cesarean delivery and complicated by cardiac arrest with successful resuscitation. Postoperative rapid uterine bleeding from DIC was treated with a Bakri balloon and NASG, stabilizing the patient during massive transfusion. Neither patient required further surgical procedures. NASG combined with Bakri balloon may serve as a valuable nonoperative treatment or temporization option in cases of massive postpartum hemorrhage complicated by coagulopathy such as these. Further study of the utility of NASG in high-resource settings is warranted.

## 1. Introduction

Disseminated intravascular coagulation (DIC) is an uncommon but potentially catastrophic complication of postpartum hemorrhage. Operative interventions, such as laparotomy for uterine compression suture placement or for hysterectomy, may increase blood loss in an already unstable patient if not preceded by rapid whole blood, platelet, and clotting factor replacement.

We describe here two cases of massive postpartum hemorrhage complicated by DIC diagnosed after completion of cesarean delivery that were successfully temporized nonsurgically with combined use of the Bakri balloon (Cook Medical, Bloomington, IN) and nonpneumatic antishock garment (NASG) (Zoex NIASG, Coloma, CA).

*Case 1: Fulminant DIC of Unclear Etiology*. A 33-year-old gravida 5 para 2 underwent labor induction at 37 6/7 weeks' gestation for gestational hypertension. Medical history was significant for sickle cell trait and history of left nephrectomy for persistent hematuria; hematologic work-up had not revealed a bleeding diathesis. The patient underwent emergent cesarean under epidural anesthesia at 5 cm dilation for sudden, persistent fetal bradycardia. Estimated blood loss was 1400 mL due to persistent oozing from the hysterotomy site requiring additional sutures to obtain hemostasis. Upon arrival in the postoperative recovery suite, brisk vaginal bleeding of nonclotting blood was noted. Bimanual exam did not suggest uterine atony, retained placenta, or lacerations. Within 10 minutes, additional blood loss had reached 1 liter and hemorrhagic shock was diagnosed (blood pressure 70/40, heart rate 140 beats per minute). The NASG was applied, followed by a Bakri balloon, while fluid resuscitation and blood transfusion ensued. Initial laboratory studies (pretransfusion) showed international normalized ratio (INR) >12.5, fibrinogen <45 mg/dL, platelets 98 × 1000/mcL, and hemoglobin 4.7 g/L. Within the first hour, the Bakri collection bag contained 350 mL blood, but output thereafter was minimal. Maternal heart rate and blood pressure improved within the first hour. A total of 8 units packed red blood cells, 6 units fresh frozen plasma, 1 pack of platelets, and 3 packs of cryoprecipitate were transfused within the first 12 hours. On postoperative day 1, maternal vital signs had normalized; INR was 1.2 and hemoglobin 7.9 g/L. The NASG and Bakri balloon were removed sequentially without incident. The patient was discharged home on postoperative day 3 in good condition.

*Case 2: Amniotic Fluid Embolism*. A healthy 31-year-old gravida 2 para 1 presented at 40 2/7 weeks in early active labor. Three hours after admission, she complained of sudden difficulty in breathing and then became unresponsive. This was followed by fetal bradycardia, and the patient was rapidly intubated for emergent cesarean delivery. Following prompt delivery of a live infant, the patient suffered bradyasystolic cardiac arrest requiring chest compressions and epinephrine. Normal sinus rhythm was restored and the surgery completed with negligible blood loss. Immediate postoperative uterine massage expressed 500 mL of nonclotting blood; intramuscular Hemabate (carboprost) and rectal misoprostol were given. On arrival to the intensive care unit, hypotension to 80/40 and tachycardia to 150 beats per minute were observed. Bimanual exam expressed two additional liters of nonclotting blood from the uterus although the uterus was not felt to be atonic. A Bakri balloon and NASG were placed while massive transfusion, intravenous fluids, and pressors were administered. Pretransfusion laboratory studies showed international normalized ratio (INR) 4.8, fibrinogen <60 mg/dL, hemoglobin 4.0 g/L, and platelets of 87 × 1000/mcL. Within the first hour, the Bakri collection bag contained 1000 mL, but bleeding diminished rapidly as DIC resolved with transfusion of a total of 14 units of packed red blood cells, 10 units fresh frozen plasma, 3 packs of platelets, and three packs of cryoprecipitate. Eight hours later, INR was 1.5 and pressors were slowly weaned. 24 hours after delivery, the Bakri balloon and NASG were removed uneventfully, and the patient was successfully extubated. On postoperative day 2, echocardiogram suggested right heart dilation, and a small upper lobe pulmonary embolus was discovered on CT angiogram. The patient was ultimately discharged on postoperative day 5 on twice daily low molecular weight heparin.

## 2. Discussion

DIC is an uncommon but potentially lethal complication of massive postpartum hemorrhage characterized by widespread activation of procoagulant activity, fibrinolysis, depletion of clotting factors, and end-organ damage. In obstetrics, inciting causes include placental abruption, postpartum hemorrhage, preeclampsia, acute fatty liver of pregnancy, sepsis, and amniotic fluid embolism [1]. Treatment of DIC includes rapid recognition, correction of inciting factors, measures to slow bleeding, if possible, and ultimately transfusion of blood, platelets, and clotting factors. For postpartum patients, bleeding can be profuse even during the interval from diagnosis to transfusion; laparotomy for hysterectomy or placement of uterine compression sutures may hasten blood loss or hemodynamic instability in the interim.

In the current case report, we describe combined use of the nonpneumatic antishock garment (NASG) and the Bakri postpartum balloon in two cases of massive postpartum hemorrhage with shock from fulminant DIC. We attribute the successful outcomes without need for further operative interventions to the combined effects of recentralization of circulating volume to treat shock and external and internal uterine compression which slowed uterine blood loss sufficiently to allow definitive treatment with fluid, blood, and clotting factor transfusion. Importantly, the combination of NASG and Bakri balloon was relatively low cost, nonoperative, and nonpharmaceutical and allowed ongoing close monitoring of maternal bleeding and hemodynamic status, without precluding the options for additional operative or medical interventions, such as recombinant factor VII, hysterectomy, or uterine artery embolization, had they been deemed necessary.

**Figure 1:**
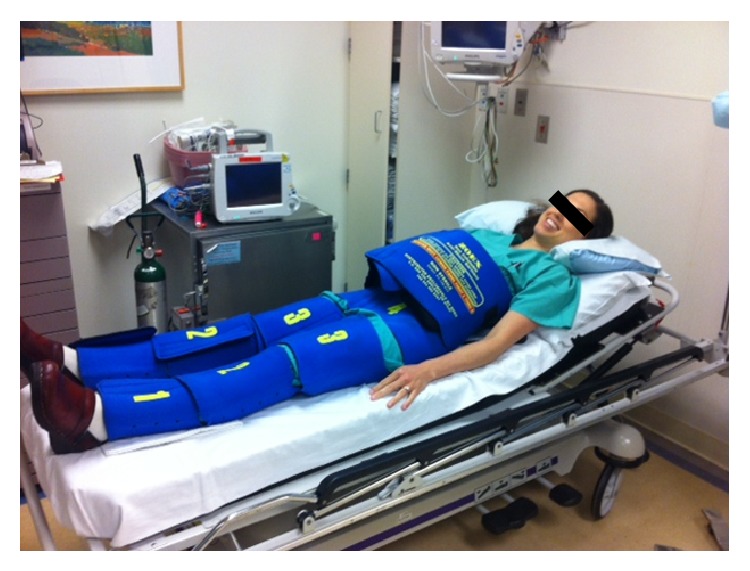
Nonpneumatic antishock garment worn in demonstration by the author (MB).

Numerous case series and several quasirandomized trials have described use of the NASG for treatment of hemorrhagic shock from massive postpartum hemorrhage, where its use in developing countries was associated with a 38% reduction in odds of maternal death from hemorrhage [2]. NASG is a segmented garment made of stretchable neoprene (see Figure 1) that is manually fastened with velcro in successive sections up the patient's two legs; the abdominal/pelvic sections contain a foam ball that additionally applies pressure to the abdominal aorta and pelvic vasculature, including the uterus. When applied, the NASG provides circumferential counter pressure at approximately 20–40 mmHg [3] aiding in centralization of circulating volume and significantly increases resistance to blood flow in the internal iliac arteries [4]. Even when fully donned, the NASG allows full access to the vagina for exams and ongoing monitoring of blood loss.

Several prior reports have described successful use of the Bakri balloon together with uterine compression sutures in the so-called “uterine sandwich” [5, 6]. In virtually all described cases, however, the patient was already in the midst of cesarean delivery, allowing ready placement of uterine compression sutures. In cases where the abdomen is not already open, including following a vaginal delivery, the decision over risks verses benefits of proceeding to laparotomy is more difficult, especially when coagulopathy is coexistent. We believe that the NASG combined with Bakri balloon may serve as a valuable nonoperative treatment or temporization option in cases of massive postpartum hemorrhage complicated by coagulopathy such as these.

Reports of NASG use in high-resource settings, where transfusion, medical and surgical treatments are generally widely available, are virtually nonexistent. Larger case series and five recent quasirandomized trials were conducted in low resource care settings in Pakistan, Nigeria, Egypt, Zambia, Zimbabwe, and India (notably without concomitant use of a Bakri balloon), where blood transfusion is either not available or significantly delayed [2, 7]. Despite promising results, NASG is not currently included in postpartum hemorrhage protocols published by the American College of Obstetricians and Gynecologists [8] or the California Maternal Quality Care Collaboration [9], and few American hospitals have NASG available for use. The success of NASG together with Bakri balloon in the current cases suggests that further study in high-resource settings is warranted, particularly whether the addition of NASG to a modern labor and delivery unit armamentarium may decrease the need for more invasive treatments or decrease costs.

In contrast to a pneumatic inflatable antishock garment, which may risk complications from overinflation, adverse outcomes reported with the use of NASG are infrequent and were found to be similar before and after NASG usage [10]. In the current report, pulmonary embolus in Case 2 was discovered on postoperative day 2; it is unclear whether this may have been a complication of the treatment or of the underlying condition. Importantly, published contraindications for NASG include congestive heart failure, mitral stenosis, pulmonary hypertension, and/or sites of bleeding above the diaphragm [3]. Close observation immediately after NASG placement is recommended, with removal indicated in cases of suspected heart failure or respiratory distress.

Published reports as well as the author's personal experience do not indicate that wearing the NASG is painful for the patient. In the current cases, pain management was not complicated by use of the Bakri and NASG together, although, in both cases, the patients were not ambulatory during this interval and had bladder catheters in place. In both cases, intrauterine location of the Bakri balloon was confirmed by ultrasound after placement of the NASG to ensure it had not been expulsed by application of the NASG; in the second case, the Bakri had to be deflated and replaced. Both devices were removed in the current cases after approximately 24 hours, which corresponded to confirmed hemodynamic stability and normalization of clotting status. In both cases, the NASG was removed first, although it seems unlikely that the order of removal would affect success rates. Removal of the NASG was performed per manufacturer's recommendations: progressively beginning with the feet sections, waiting 15 minutes for equilibration between sections, and monitoring pulse and blood pressure closely.

In summary, NASG combined with Bakri balloon was successfully employed in two cases of massive postpartum hemorrhage complicated by DIC. Further study of the utility of NASG in high-resource settings is warranted.
